# The second terminations of the suboccipital muscles: An assistant pivot for the To Be Named Ligament

**DOI:** 10.1371/journal.pone.0177120

**Published:** 2017-05-25

**Authors:** Xiao-Ying Yuan, Chan Li, Jia-Ying Sui, Qi-Qi Zhao, Xiao Zhang, Na-Na Mou, Zhao Huang-Fu, Okoye Chukwuemeka Samuel, Nan Zheng, Seung-Ho Han, Sheng-Bo Yu, Hong-Jin Sui

**Affiliations:** 1 Department of Anatomy, College of Basic Medicine, Dalian Medical University, Dalian, China; 2 The second clinical college, Dalian Medical University, Dalian, China; 3 The first clinical college, Dalian Medical University, Dalian, China; 4 Department of Anatomy, College of Medicine, Chung-Ang University, Seoul, Korea; Chang Gung University, TAIWAN

## Abstract

In the last two decades, many studies have focused on the muscles and dense connective tissues located in the suboccipital region. Our study investigated the existence of the second terminations originating from the suboccipital muscles, and the relationship between the variable types of the To Be Named Ligament (TBNL). Anatomical dissection was performed on 35 head-neck specimens. The existence of the second terminations of the suboccipital muscles was confirmed and various types of the TBNL were observed in this study. The second terminations originated from multiple suboccipital muscles including the rectus capitis posterior minor (RCPmi), rectus capitis posterior major (RCPma) and obliquus capitis inferior (OCI) muscles, merged and terminated at the TBNL. The overall incidence of the second terminations of the suboccipital muscles was 34.29% and it varied among the various suboccipital muscle origins. 28.57% of the second terminations originated from the RCPma; 11.43% was from the RCPmi and 8.57% was from the OCI. Furthermore, there was a significant relationship between the existence of second terminations and the particular type of the TBNL. 95% of the arcuate type of the TBNL was accompanied with the second terminations which attached to their turning part, whereas only 10% of all the radiate type of the TBNL was accompanied with the second terminations. This study for the first time described the second terminations originating from multiple suboccipital muscles and demonstrated the relationship with the various types of the TBNL. We speculated that the second terminations maintain the arcuate TBNL and transfer tensile forces to the Myodural Bridge (MDB), thereby modulating the physiological functions of the MDB.

## Introduction

The suboccipital region is one of the most complicated anatomical areas of the human body [1]. In 1995, Hack et al. first described the relationship between the deep suboccipital muscles and the cervical spinal dura mater. They found a connective tissue bridge named the Myodural Bridge (MDB), between the rectus capitis posterior minor (RCPmi) and the dorsal cervical spinal dura mater at the atlanto-occipital interspace. The RCPmi gave off dense connective tissue that connects with the posterior atlanto-occipital membrane, and finally merged with the dorsal cervical spinal dura mater [2]. To date, many studies have confirmed the existence of this connective tissue bridge in humans and other mammalian animals [3]. Moreover, many studies in the last decade have shown that the MDB originate from multiple suboccipital muscles including the rectus capitis posterior major (RCPma) and the obliquus capitis inferior (OCI) [4–13].

In the suboccipital region, the nuchal ligament (NL) also provides a connection between the suboccipital region and the cervical dura mater [14–17]. In 2014, we observed an intrinsic fascial structure called the To Be Named Ligament (TBNL). The TBNL is a dense fibrous band that originates from the lower part of the posterior border of the NL, runs anteriosuperiorly to enter the atlanto-axial interspace and merged with the posterior cervical dura mater. It thereby forms part of the MDB. Furthermore, the TBNL is formed by either arcuate fibers or radiate fibers. In this study, we also found a second termination which originated from the RCPmi and terminated at the arcuate fiber of the TBNL [17].

Here in this research, we conducted an extensive anatomical study about the deep suboccipital region, and we found that multiple suboccipital muscles (RCPmi, RCPma and OCI) had novel terminations on the TBNL other than the traditional bony structures. We termed these novel terminations as the “second terminations” and we additionally investigated the morphological relationship between these second terminations and the variable types of the TBNL in the deep suboccipital region of humans.

## Materials and methods

### Dissection of the suboccipital muscles

Thirty-five donated adult head-neck specimens (23 males, 12 females) were dissected in this study. All the specimens were obtained from the Department of Anatomy, Dalian Medical University. This study was approved by the Ethics Committee of the Body and Organs Donation Center of Dalian Medical University. None of the tissue donors were from a vulnerable population and all donors or next of kin provided written informed consent that was freely given.

The specimens were preserved using a formalin-alcohol mixture. A layer-by-layer dissection was performed to expose the suboccipital muscles, the NL as well as the TBNL. The second terminations of the RCPma and OCI were observed and documented, and we subsequently cut the origin of the RCPma from the inferior nuchal line to expose the RCPmi. The second termination of the RCPmi was then observed and documented. Following these procedures, the various suboccipital muscle originating second terminations and the interrelationship with neighboring structures were observed. Photographic documentations were recorded with a Canon EOS 60D camera.

### Observation of the second terminations and the TBNL

The second termination was defined as bundle of muscle fibers that originated from the RCPmi, the RCPma and the OCI, and terminated at the soft tissue structures (TBNL) instead of bony structures, e.g. the posterior tubercle of atlas or spinous process of axis. We observed and counted the existence of the second terminations in all the thirty-five specimens.

The TBNL had two types of fiber arrangements, the arcuate and the radiate fibers. The arcuate fibers of the TBNL originated from the lower part (below vertebra C3) of the posterior border of the NL, ran anterosuperiorly; crossed over the spinal process of the axis and continued into the atlanto-axial interspace. The radiate fibers of the TBNL arose from the upper part of the NL (above vertebra C3), ran anteriorly and straightly into the atlanto-axial interspace, and finally attached to the cervical dura mater.

### Statistics

For statistical analysis, *chi-square* test was applied (SPSS 17.0, IBM, USA). A *P* value smaller than 0.05 indicated statistically significance. No specimen was excluded in the analysis.

### Ethics statement

The body and organs donation Center of Dalian Medical University is a specialized office that accepts body and organs from volunteers and donors' body for scientific research and medical education. It has a strict system of accepting donated body. These bodies and organs received by this center are by the agreement of both the donors and their immediate families. The specimens used in the experiment of this article “The Second Terminations of the Suboccipital Muscles: an Assistant Pivot for the To Be Named Ligament” by Xiao-Ying Yuan et al. were all from donations of the volunteers to the donation center and were all legally collected. The donation center allowed these specimens to be used in this basic medical research. None of the tissue donors were from a vulnerable population and all donors or next of kin provided written informed consent that was freely given.

This can be verified in the website below.

URL: http://home.dlmedu.edu.cn/bodc/

## Results

In the suboccipital region, the paired RCPmi and RCPma were short muscles and the RCPmi lied ventrally to the RCPma. The RCPma originated from the pcciput and the RCPmi originated from the position between the inferior nuchal line of the occiput and the foramen magnum, descended to attach at the posterior tubercle of the atlas and the spinous process of axis respectively. The OCI-originated from the transverse process of atlas and descended medially to attach at the spinous process of the axis.

### Existence and incidence of the second terminations from the suboccipital muscles

**Fig 1:**
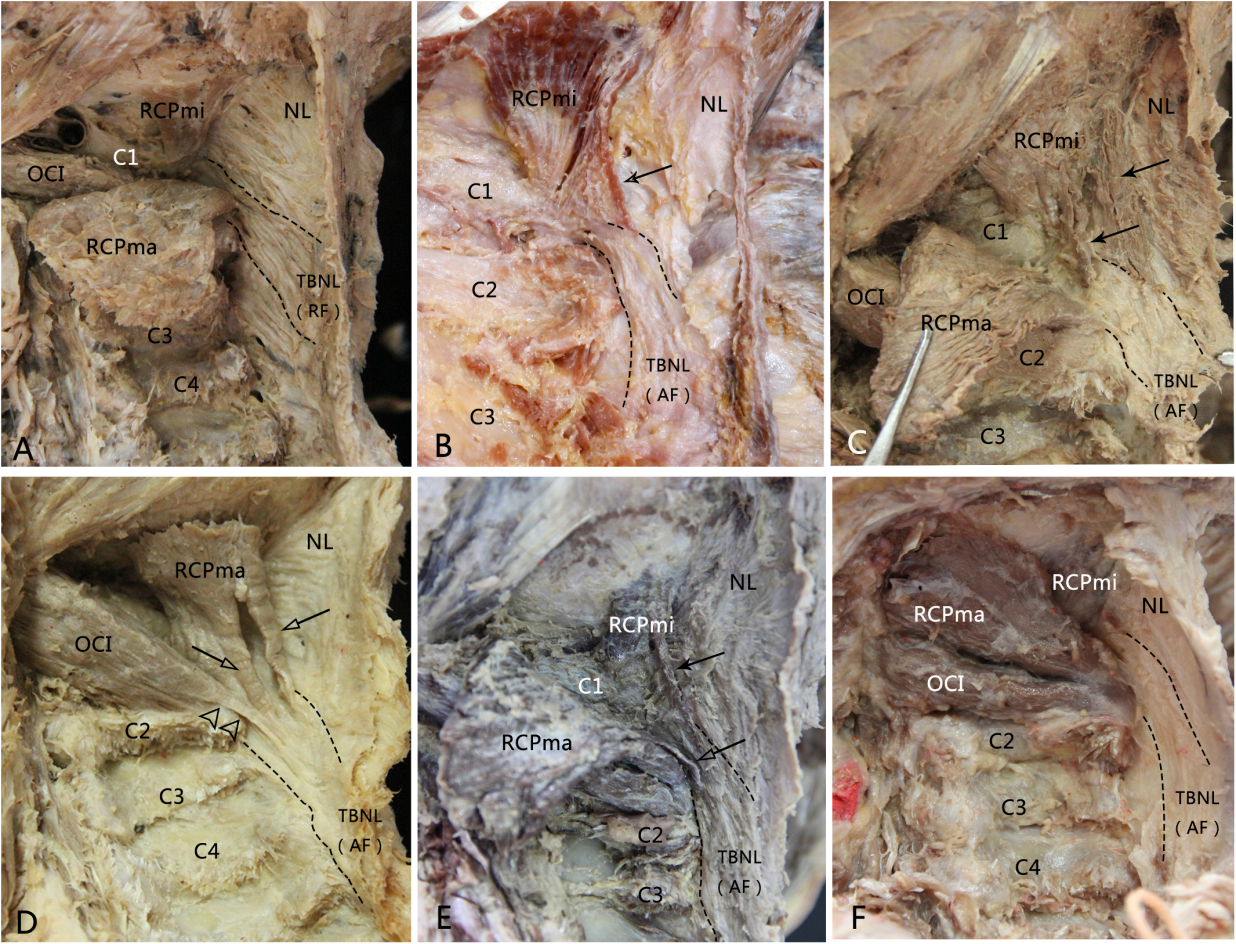
The second terminations of the suboccipital muscles (RCPmi, RCPma and OCI) and the types of the TBNL in the dissected specimens. A, The TBNL within the NL was formed by radiate fibers. Opposite to the spinal processes of vertebrae C2 and C3, the radiate fibers originated from the posterior border of the upper part of NL, ran anterosuperiorly and straightly entered into the posterior atlanto-axial interspace. No second termination was found from the RCPmi, RCPma and OCI. B, The TBNL within the NL was formed by arcuate fibers. The arcuate fibers arose from the lower part of the posterior border of NL below the level of the spinal process of C3, ran anterosuperiorly, crossed over the spinous process of axis to continue into the posterior atlanto-axial interspace. A muscle bundle of the RCPmi separated and terminated at the level of atlanto-axial interspace. C, The RCPmi emitted multi-bundles of muscular fibers and attached to the arcuate TBNL at the level of the posterior arch of atlas and the axis. D, The TBNL was arcuate and the second terminations originated from the RCPma and the OCI. The OCI emitted a tendinous bundle which crossed behind the spinous process of the axis and continued with the arcuate TBNL. Two muscular bundles of the RCPma terminated at the TBNL at the level of the posterioratlanto-axial interspace. E, The TBNL was arcuate and the second terminations of the RCPmi and the RCPma were existed simultaneously. F, The arcuate TBNL was accompanied without a second termination of the suboccipital muscles. RF: radiate fibers; AF: arcuate fibers; Arrow: second termination of the RCPmi; Hollow arrow: second termination of the RCPma; Double hollow triangles: second termination of the OCI.

In our study, we found novel terminations of the suboccipital muscles, and we termed them as the second terminations. These second terminations originated from the suboccipital muscles and terminated at the TBNL, and the overall incidence of the second terminations was 34.29% (24 out of 70 sides). In these 24 sides, the RCPma-originated second terminations were found in 20 sides and had the greatest incidence rate of 28.57% (Fig 1D and 1E); 8 sides showed the RCPmi-originated second terminations, the incidence was 11.43% (Fig 1B, 1C and 1E); the OCI-originated second terminations were found in 6 sides, the incidence was 8.57%, which was the lowest (Fig 1D).

The suboccipital muscles gave off muscle bundles of the second terminations near the midline and these muscle bundles terminated at the TBNL, just before the TBNL entered the posterior atlanto-axial interspace. Second terminations of the RCPmi and RCPma were either composed of one (Fig 1B) or multiple (Fig 1C and 1D) muscle bundles. The fiber property of the OCI-originated second terminations was tendinous, which was more resistant to tensile forces, compared with the muscular second termination of RCPmi and RCPma (Fig 1D).

In some cases, the second terminations originated at the same side from multiple suboccipital muscles and terminated at the TBNL. The second terminations originating from both the RCPma and the OCI were observed in 4 sides (Fig 1D); those from both the RCPmi and the RCPma were found in 6 sides (Fig 1E, Table 1).

**Table 1:** The relationship between the second terminations of the RCPmi, RCPma and OCI and the types of the TBNL.

Second termination	origin	TBNL		total
		Radiate	Arcuate	
Existence	RCPmi	0	8 (6^▲^+2)	
	RCPma	5 (4+*1*^★^)	15 (3*+6^▲^+6)	24
	OCI	*1*^★^	5(3*+2)	
Non-existence		45	1	46
Total		50	20	70
*P* Value		<0.001☆		

Note

^★^: Second terminations of the RCPma and the OCI co-existed on 1 side.

^▲^: Second terminations of the RCPmi and the RCPma co-existed on 6 sides.

*: 3 sides had the second terminations from the RCPma and the OCI.

☆: *chi-square* test

### The relationship between the second terminations and the types of the TBNL

The TBNL was a dense fibrous band that originated from the posterior funicular portion of NL, ran anteriorly through the atlanto-axial interspace and attached to the posterior aspect of the cervical dura mater. It formed part of the MDB. The TBNL was formed by either arcuate or radiate fibers according to the point of origin of the fiber tissues [17].

The radiate type of the TBNL was found in 50 out of 70 sides (Fig 1A) with the incidence of 71.43%. Twenty of seventy sides had the arcuate type of TBNL (Fig 1B, 1C, 1D, 1E and 1F) with an incidence of 28.57%. In 20 of 70 sides with the radiate type of the TBNL, 19 sides had the second termination: 2 sides from RCPmi only, 6 sides from the RCPma only, 2 sides from the OCI only; 6 sides had the second terminations simultaneously derived from the RCPmi and RCPma (Fig 1E), and 3 sides had second terminations from both the RCPma and the OCI (Fig 1D). In 50 of 70 sides with the radiate type of the TBNL, only 5 sides had the second termination: 1 side from the RCPma, 4 sides from both the RCPma and OCI. There was no existence of second termination in the other 45 sides (Table 1). The relationship between the second terminations of suboccipital muscles and the types of the TBNL was statistically significant (P<0.001).

Since we conducted the gross anatomy on 35 head-neck specimens, we picked up the best photographs and put them as figures in the manuscript; see S1 Figs for the second terminations and their neighboring structures of some other head-neck specimens.

## Discussion

### The existence of second terminations of the suboccipital muscles

Traditionally, the origins of RCPmi and RCPma originate from the inferior nuchal line and the occiput, and the terminations of these two suboccipital muscles are at the posterior tubercle of the atlas (RCPmi) and spinous process of axis (RCPma), respectively. The OCI originates from the transverse process of atlas and descends to the medial spinous process of the axis. In this study, we found new terminations of the suboccipital muscles of RCPmi, RCPma and OCI to the TBNL and we termed these new findings as the second terminations.

Based on the results of the gross anatomy, muscular or tendinous bundles separated from the suboccipital muscles (RCPmi, RCPma, OCI), ran along with the NL in the midline and finally terminated at the TBNL. The second terminations may originate from multiple suboccipital muscles, and the overall incidence of these second terminations was 34.29%. The RCPma-originated second termination showed the highest incidence (28.57%) according to our observation. None of these findings have been reported in any previous publications.

### Relationship between the second terminations and the arcuate TBNL

The TBNL is first described and defined in 2014 [17]. It is a dense fibrous band, intrinsic component and enhancement of the NL that forms part of the MDB. It enters the epidural space through the atlanto-axial interspace and terminates at the posterior cervical spinal dura mater. In this study, we found a significant correlation between the appearance of the second terminations and the types of the TBNL. In the 20 sides of the arcuate TBNL, the incidence of the second terminations of the suboccipital muscles was 95% (19/20), while in 50 sides of the radiate TBNL, the incidence of second terminations was 10% (5/50).

In this study, we speculatively conclude that the second terminations usually co-exist with the arcuate TBNL. The arcuate TBNL originates from the posteroinferior part of the NL, runs anterosuperiorly crossing over the spinous process of the axis, and continues ventrally into the posterior atlanto-axial interspace. The existence of the second terminations would optimize the functional performance of the arcuate TBNL. The forces of contraction of the RCPmi, RCPma and OCI may act directly through the second terminations to maintain the TBNL, which curve around the posterior part of the spinous process of the axis, thus acting as an assisting pivot for the TBNL. Additionally, the second terminations might collaborate with the arcuate TBNL to transfer forces to the MDB via the atlanto-axial interspace. This will greatly enhance the physiological functions of the MDB [18]. To this hypothesis, analysis of the biomechanical coordination between the second terminations of the suboccipital muscles and the arcuate TBNL will be our next research target.

## Supporting information

S1 Figs The photographs about the second terminations and their neighboring structures of some other head-neck specimens during the gross anatomy. Since we conducted the gross anatomy on 35 head-neck specimens, and we picked up the best photographs and put them as figures in the manuscript; these photographs are about the second terminations and their neighboring structures of some other head-neck specimens. None of structures was labeled.
(RAR)
